# Estimation of Leaf Nitrogen Content from Spectral Characteristics of Rice Canopy

**DOI:** 10.1100/tsw.2001.387

**Published:** 2001-12-19

**Authors:** Chwen-Ming Yang

**Affiliations:** Department of Agronomy, Taiwan Agricultural Research Institute, Wufeng, Taichung Hsien, China

**Keywords:** leaf nitrogen content, spectral characteristics, spectral reflectance, rice canopy, hyperspectral resolution

## Abstract

Ground-based remotely sensed reflectance spectra of hyperspectral resolution were monitored during the growing period of rice under various nitrogen application rates. It was found that reflectance spectrum of rice canopy changed in both wavelength and reflectance as the plants developed. Fifteen characteristic wavebands were identified from the apparent peaks and valleys of spectral reflectance curves, in accordance with the results of the first-order differentiation, measured over the growing season of rice. The bandwidths and center wavelengths of these characteristic wavebands were different among nitrogen treatments. The simplified features by connecting these 15 characteristic wavelengths may be considered as spectral signatures of rice canopy, but spectral signatures varied with developmental age and nitrogen application rates. Among these characteristic wavebands, the changes of the wavelength in band 11 showed a positive linear relationship with application rates of nitrogen fertilizer, while it was a negative linear relationship in band 5. Mean reflectance of wavelengths in bands 1, 2, 3, 5, 11, and 15 was significantly correlated with application rates. Reflectance of these six wavelengths changed nonlinearly after transplanting and could be used in combination to distinguish rice plants subjected to different nitrogen application rates. From the correlation analyses, there are a variety of correlation coefficients for spectral reflectance to leaf nitrogen content in the range of 350-2400 nm. Reflectance of most wavelengths exhibited an inverse correlation with leaf nitrogen content, with the largest negative value (r = –0.581) located at about 1376 nm. Changes in reflectance at 1376 nm to leaf nitrogen content during the growing period were closely related and were best fitted to a nonlinear function. This relationship may be used to estimate and to monitor nitrogen content of rice leaves during rice growth. Reflectance of red light minimum and near-infrared peak and leaf nitrogen content were correlated nonlinearly.

## INTRODUCTION

The site-specific aspects are the primary concern of precision agriculture. One of the goals of precision farming is to match nitrogen (N) supply with crop requirement at any point in a field. This goal requires spatial information on the N status of both crop and soil. N deficiency is a universal issue in crop production, and its symptom expression is fairly uniform throughout the vegetation canopy. Therefore, it is possible to estimate plant N content from the spectral data of light reflection [1,2,3,4,5,6,7,8,9]. The hypothesis is mainly based on the relationship between N and leaf area development and between N content and chlorophyll content[5,10].

N status or N availability of plant tissue and the soil can be measured chemically or physically. Chemical methods require considerable effort for sample collection and processing, making repeated sampling throughout the growing season labor intensive and time consuming. Physical approaches are the other options, which provide nondestructively instantaneous results of measurements. In particular, the spectral remote-sensing technology has the capability of sampling and determining biological and physical properties of a plant community or a crop canopy rather than of a single plant or a single spot of vegetation[6,7,10,11,12,]. Consequently, the technology could provide a quick way to assess the spatial and temporal variability within a field. To apply N fertilizer at proper rates, the spatial variation of soil mineral N that will become available over the growing season must be properly assessed. Also, N content in leaves and crop canopy should be estimated appropriately.

The objectives of this research were to study N status of rice leaves under different N application rates using ground-based canopy reflectance of hyperspectral resolution. The spectral characteristics of rice canopy suitable for leaf N content estimation were retrieved, and the relationships between these variables were determined.

## EXPERIMENTAL METHODS

Field experiments for ground-based rice spectral measurements were carried out at the experimental farm of Taiwan Agricultural Research Institute, Wufeng (24°45'N, 120°54'E, elevation of 60 m), during the growing season of 2000, from August 7 to December 8. The experimental plot was a loamy soil with pH of 5.60 and was divided into six 50 m × 15 m subplots. The 15-day-old rice seedlings (*Oryza sativa* L. cv. Tainung 67) were machine-transplanted to the field in a north-south direction at a density of 22 hills per square meter with row spacing of 0.3 m and plant distance of 0.15 m. The chemical properties of the soil at transplanting were as follows: OM(spell out OM): 1.2%, Kjeldahl-N: 755 ppm, available phosphorus: 9.5 ppm, exchangeable potassium: 44.3 ppm, exchangeable calcium: 623 ppm, and exchangeable magnesium: 107 ppm.

N fertilizer ammonium sulfate was applied to the experimental plot at rates of 0 (carbon and potassium), 30, 60, 90, 120, and 150 kg $ha^{-1}$, with each quantity sprayed as three equal dressings on August 16, September 8, and October 2, 2000, respectively. Rice plants were grown at different N levels in order to obtain a range of reflectance spectra. N determinations were conducted on the days of spectral measurements during rice growth. Six plants were collected on each subplot, and leaf N content was determined by the Kjeldahl method. The pesticides were applied as needed and recommended, and irrigation water was well-controlled to avoid any stress impact.

Canopy spectral measurements began on September 5, 29 days after transplanting (DAT) and were made periodically until December 7, the day before harvest. By comparing the spectral waves, most parts of reflectance spectrum were found to be unaffected by background affects after 29 DAT where the crop canopy covered more than 50% of the ground area. Reflectance spectrum was taken on clear or partly cloudy days by a field portable spectroradiometer (model GER-2600, Geophysical & Environmental Research Corp., Millbrook, New York, USA), which has a field of view of 10 degrees and was mounted about 5.8 m above the rice canopy at nadir. The GER-2600 uses a fixed grating array-based design, splitting into three spectrometers. One spectrometer incorporates a 512-element silicon detector array that measures a wavelength range of 300 nm to 1050 nm with a sampling interval of 1.5 nm. The other spectrometers utilize a 128-PbS (spell out) detector array measuring a wavelength range of 1050 nm to 2500 nm with a sampling bandwidth of 11.5 nm. As the spectra above 2400 nm were very noisy and seemed not to affect the objectives of this study, data obtained above 2400 nm were omitted from analysis. At every measurement time, at least 15 spots of measurements per subplot were taken. Each measurement set had a mean of four individual full-range (350-2500 nm) spectral scans. The mean reflectance spectrum was calculated from the 15 measurements per subplot. Reflectance spectrum was obtained by comparing radiance of the target canopy to radiance of the spectral reference panel (Spectralon, Labsphere, Inc., North Sutton, New Hampshire, USA).

The spectral characteristics of canopy spectrum were studied as follows. For each N treatment, 15 wavelengths were determined by the first-order differentiation in accordance with the apparent turning points (peaks and valleys) that appeared in the average reflectance spectrum on each measuring day. The 15 wavelength intervals grouped from the growing season were named the characteristic wavebands (CWs), and the center wavelength of every waveband and its standard deviation were calculated. The mean reflectance and standard deviation of the individual center wavelengths were also computed. Correlations between wavelength and reflectance of the center wavelengths and N application rates were determined. The correlation intensity of spectral reflectance to leaf N content was analyzed in the measured spectral domain at different N treatments as well as in pooled data. Wavelengths in red light minimum (RED) and the near-infrared (NIR) peak were determined, and wavelength with the maximum correlation coefficient was acquired. The relationships of these spectral characteristics to leaf N content were studied. Relationships between the normalized difference vegetation index (NDVI) and the RED/NIR ratio and leaf N content were also analyzed. The NDVI was calculated by the formula (NIR - RED) / (NIR + RED), where NIR is the reflectance of the near infrared peak and RED is the reflectance of the red light minimum.

## RESULTS AND DISCUSSION

Knowing the relationship between spectral characteristics of crop canopy and crop N status is essential for site-specific management of N fertilizer. The relationship can be extracted from field experiments in which various N application rates were applied. In this study, the ground-based remotely sensed reflectance spectrum data, from 350 to 2400 nm, for rice canopy under various levels of N fertilizer applications rates were measured and monitored using a portable spectroradiometer. As indicated in Fig. 1, persistent changes in spectral reflectance were observed with developmental age. The reflectance spectra were also influenced by the environmental affects. The simplified features of spectral curves, the so-called spectral signatures, for different N treatments were obtained by connecting the wavelengths of 15 CWs (Fig. 2). These 15 wavelengths varied during the rice growth (Fig. 3) and had different reflectance (Fig. 2). By viewing the spectral signatures, the progress of reflectance spectrum can be easily seen in overview, and the changes can then be readily compared. However, spectral signature was not constant with plant

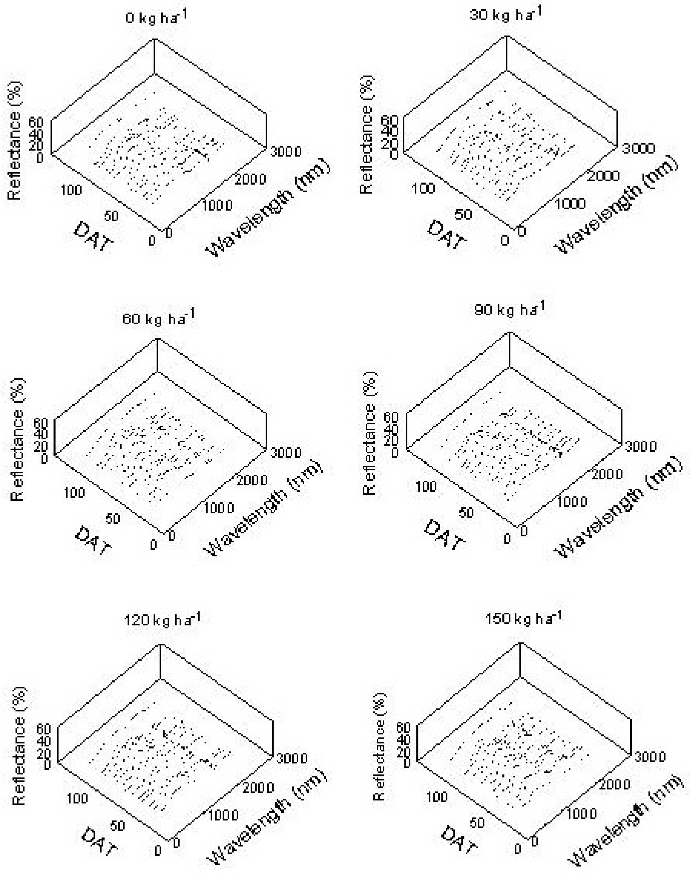

**FIGURE 1.** Changes in canopy reflectance spectrum (350–2400 nm) of rice plants grown under different nitrogen fertilizer application rates (0, 30, 60, 90, 120, and 150 kg ha$^{-1}$) during the growing season of 2000.

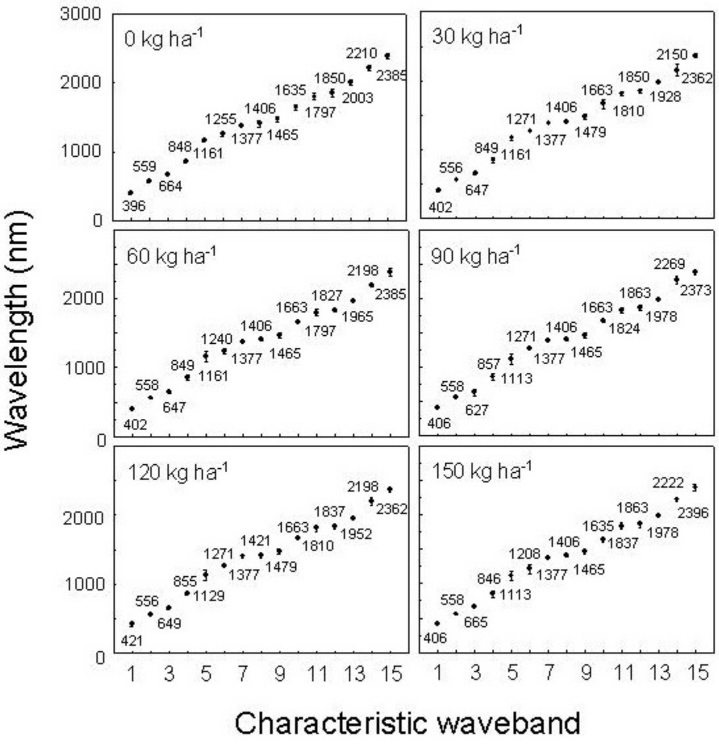

**FIGURE 2.** The center wavelengths and standard deviations of 15 characteristic wavebands selected from canopy reflectance spectra of rice plants grown under different nitrogen fertilizer application rates (0, 30, 60, 90, 120, and 150 kg ha$^{-1}$) during the growing season of 2000.

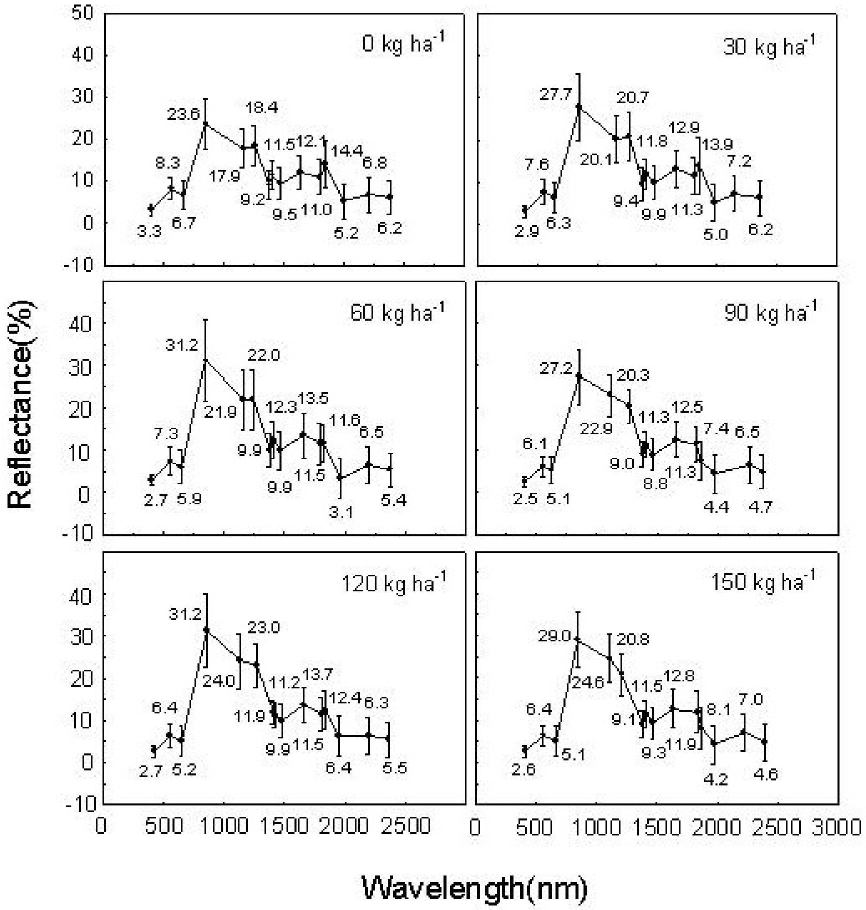

**FIGURE 3.** The mean reflectance and standard deviations of 15 characteristic wavebands selected from canopy reflectance spectra of rice plants grown under different nitrogen fertilizer application rates (0, 30, 60, 90, 120, and 150 kg ha$^{-1}$) during the growing season of 2000.

development due to the alteration of plant structure and pigmentation as well as the variations in N supply and environmental conditions. This is expected because the mixed signal of canopy spectrum consists of leaves, culms, and panicles and is vulnerable to disturbance from external factors.

Among these 15 CWs, change of the wavelength in band 5 showed a negative linear relationship with application rates of N fertilizer, while it was a positive linear relationship in band 11 (Fig. 4). Changes of wavelengths in band 5 at 28 DAT and in band 11 at 73 DAT and 100 DAT were closely correlated with N application rates (Fig. 5). Mean reflectance of the wavelengths in bands 1, 2, 3, 5, 11, and 15 were also significantly correlated with N application rates (Fig. 4). Reflectance of these six wavelengths changed nonlinearly after transplanting (data not shown), and the coefficients of correlation with N application rates may be significant in some stages but not in others during the growing period (Fig. 6). These data suggest that CWs in relation to different N application rates or N impact are time dependent. This is no surprise since the reflectance spectrum is made up of the confounded affects of plant structure, population architecture, and pigmentation response. These affects alter during the plant development and are affected by external factors such as sun angle, atmospheric conditions, climatic fluctuation, and fertilizer application. Therefore, the correlation coefficients are unstable during rice growth, and one waveband may be applicable to certain growth stages but not to the whole growing period. Such facts limit the use of a single waveband to determine N affect. Nevertheless, the use of spectral behavior of multiple wavebands may still be able to distinguish rice plants subjected to different N treatments, in excess or in deficiency.

The correlation intensity curves of spectral reflectance to leaf N content in the range of 350-2400 nm among different N application rates were compared in Fig. 7. The data show that there are a variety of correlation coefficients along the measured spectral domain. These intensity curves provide a whole picture of the correlation profile in the wavelength axis and a comparison of rice plants that received different rates of N fertilizer. The data also demonstrate that change of spectral reflectance is related to the leaf N content of rice plants. From ultraviolet (350-400 nm) to middle infrared (1800-2400 nm), most wavelengths exhibited an inverse correlation with leaf N content, with the largest negative value ($r$ = -0.581) located at about 1376 nm. That is, reflectance at 1376 nm provides the most sensitive estimation of leaf N content compared to other wavelengths. By plotting the data, the relationship between reflectance and leaf N content at 1376 nm was best fitted to a nonlinear function with a coefficient of determination ($R^2$) of 0.416 (Fig. 8).

It is well-known that chlorophyll has strong absorption bands in both blue- and red-light regions[13,14], but the bands will be shifted owing to the changes of chlorophyll concentration, decomposition of chlorophyll, and pigment proportion when the plant aged[15,16,17,18,19] or under environmental stress[2,14,20]. Because the majority of leaf N was incorporated into chlorophylls, different levels of N in rice leaves will cer-

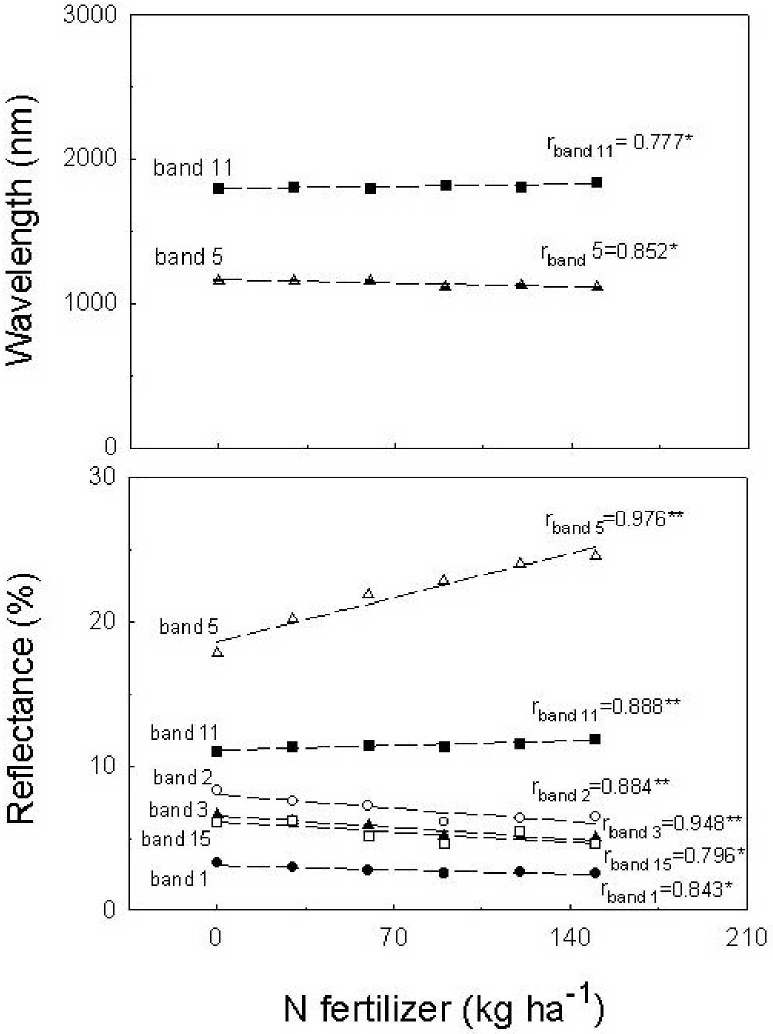

**FIGURE 4.** Wavebands and spectral reflectance identified from rice canopy reflectance spectra can be used to distinguish the affects of different nitrogen fertilizer application rates.

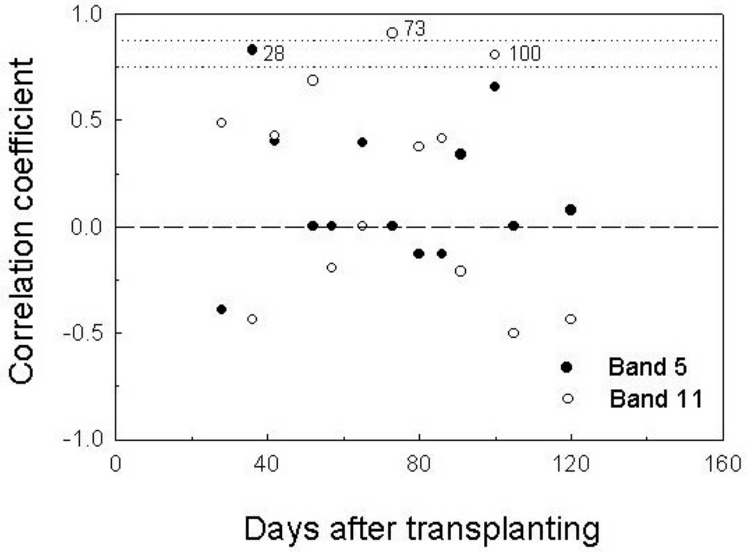

**FIGURE 5.** Temporal changes of correlation coefficients between wavelengths of band 5 and band 11 and nitrogen fertilizer application rates after transplanting.

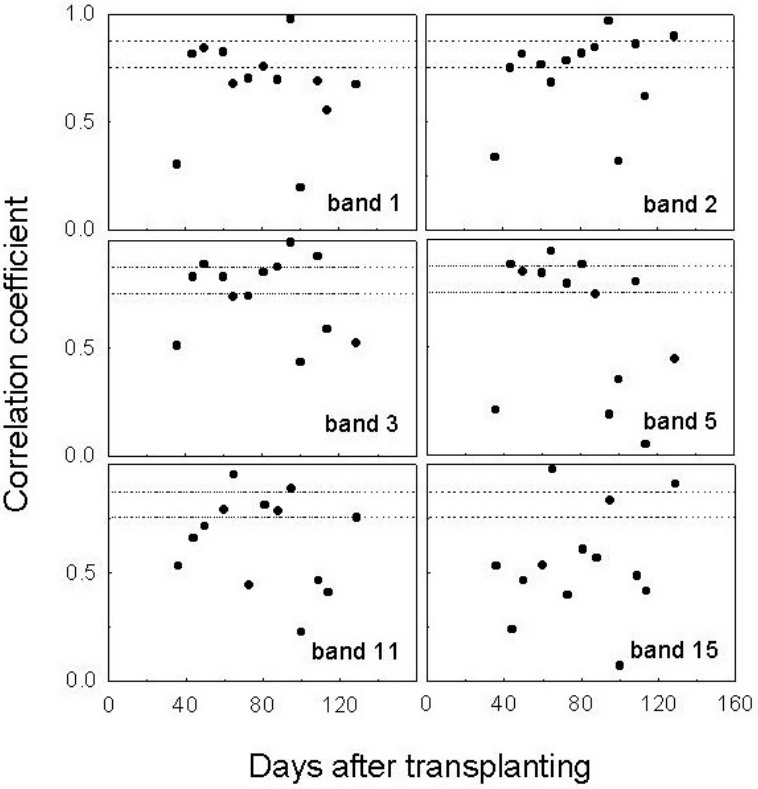

**FIGURE 6.** Temporal changes of correlation coefficients between reflectance of bands 1, 2, 3, 5, 11, and 15 and nitrogen fertilizer application rates after transplanting.

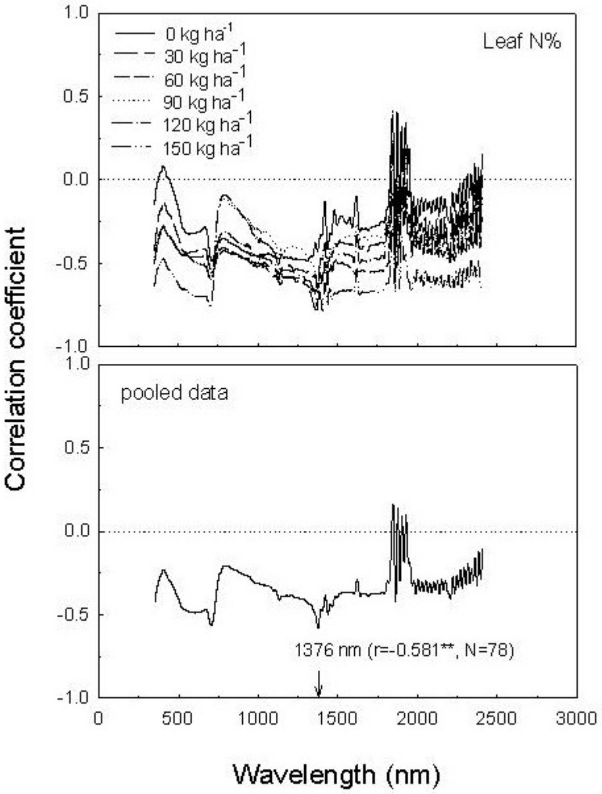

**FIGURE 7.** The correlation intensity curves of spectral reflectance to leaf nitrogen content in the range of 350-2400 nm in rice plants grown under different nitrogen fertilizer application rates (0, 30, 60, 90, 120, and 150 kg $ha^{-1}$) during the growing season of 2000.

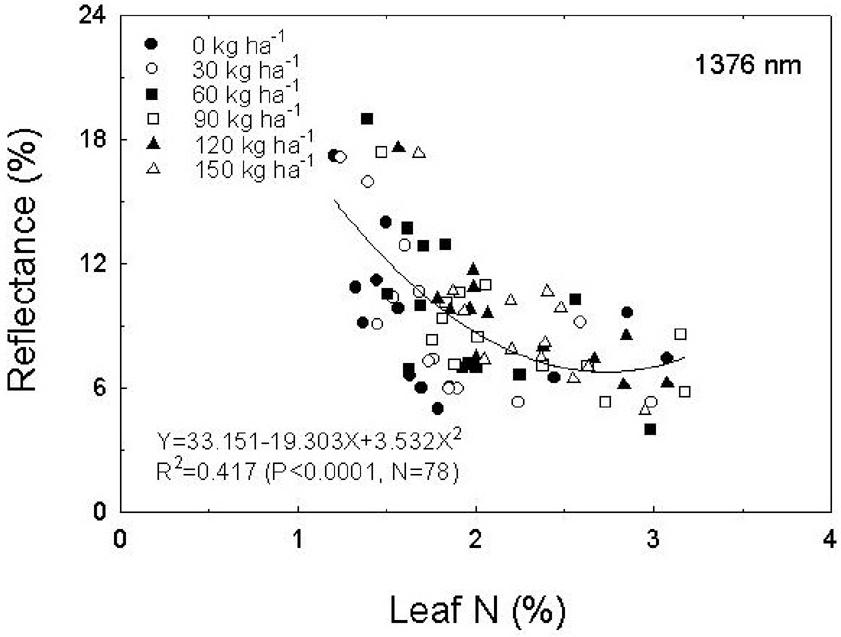

**FIGURE 8.** Correlation between reflectance and leaf nitrogen content at wavelength of 1376 nm in rice plants grown under different nitrogen fertilizer application rates (0, 30, 60, 90, 120, and 150 kg ha$^{-1}$) during the growing season of 2000.

tainly affect reflectance spectrum of rice canopy. Differences in vegetation cover, biomass, canopy geometry, and water content due to the N treatments may also contribute to the variations in spectral reflectance measurements. The sensitive response in reflectance at 1376 nm may be the mixed result of confounding affects that need to be further clarified and differentiated.

Correlations between reflectance of RED and NIR and leaf N content were correlated nonlinearly (Fig. 9), with less satisfactory results for NIR ($R^2 = 0.171$). Changes in vegetation indices of RED/NIR ratio and NDVI to leaf N content were also closely related. The determination coefficients were 0.468 and 0.487 for RED/NIR ratio and NDNI, respectively. The regression equa-

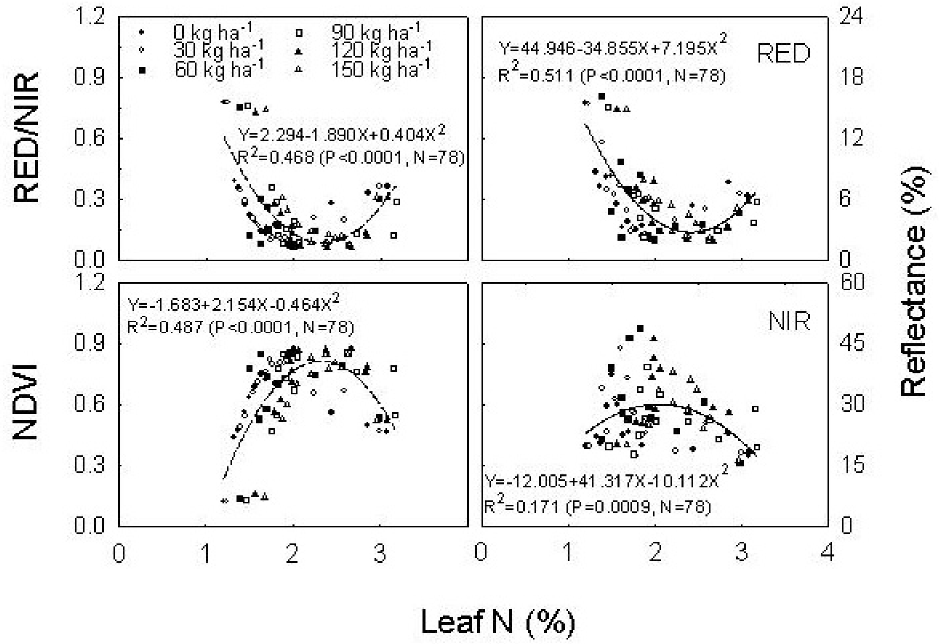

**FIGURE 9.** Correlation between reflectance at the red light minimum (RED), near-infrared peak (NIR), vegetation indices (RED/NIR ratio and NDVI), and leaf nitrogen content in rice plants grown under different nitrogen fertilizer application rates (0, 30, 60, 90, 120, and 150 kg ha$^{-1}$) during the growing season of 2000.

tions with reflectance or vegetation index as a function of leaf N content were also provided, by which leaf N content of field-grown rice may be estimated and monitored from these spectral characteristics. The estimation of the leaf N content using these equations worked well in the range of 1%-4%. As vegetation indices are mostly sensitive to canopy characteristics[10] rather than to water content, chemical compounds, or physicophysiological interactions, no doubt there is a limit of vegetation indices for assessment of plant physiological function. It is therefore not unexpected to have a lesser degree of correlation with leaf N content, which confines the potential for developing techniques for the variable-rate application of N fertilizer to agricultural fields.

## CONCLUSIONS

N is one of the nutrients having the largest affect on the growth and yield of cereal crops. However, N availability in the soil is very dynamic, not only between seasons but also within a season due to variability in mineralization, leaching, denitrification, and crop N uptake[2]. Therefore, precision in timing and in the amount of N application seems to be needed to optimize the crop yield and product quality. Currently, there has been much attention on applying variable-rate technology for N fertilizer because of the environmental and financial benefits[21,22]. Remote-sensing data allow for estimating the site-specific N uptake and for predicting the amount of N fertilizer required in a site-specific farming system.

Experimental results from Thomas and Oerther[8] showed that leaf reflectance could be used to quickly estimate the N status of sweet peppers. Differences of N treatments in corn and wheat were demonstrated and may be detected from spectral reflectance data[6,23,24,25]. It was found that the ratio of reflected radiation in the wavebands of 550-600 nm and 800-900 nm could be used to detect N stress in corn[2]. Results of this study also indicate that the spectral remote-sensing technique has the potential to evaluate the N status of rice leaves. However, at this point, leaf N content of rice can be estimated only roughly by the canopy spectral characteristics identified in this study. Moreover, as this study was conducted on a single experimental field with only one genotype of rice, the influences of different varieties, soil types, and climatic conditions on crop N status need further investigation. Such information is required for generating a canopy N content map and for producing an N application map. Additional experiments and analyses are ongoing to obtain more data to improve the relationships and to compare these spectral characteristics with the aboveground N content (canopy N) and physical traits.

## ACKNOWLEDGMENTS

This work was supported by research grants (89AST-1.7-IC-01Z(1) and 90SA-1.4-IC-01(09)) from Council of Agriculture, the Executive Yuan, Taiwan (ROC).

| | | |
|---|---|---|
| Received: | July | 27, 2001 |
| Revised: | November | 11, 2001 |
| Accepted: | November | 12, 2001 |
| Published: | December | 19, 2001 |

## BIOSKETCH

**Chwen-Ming Yang**, Ph.D., is an Agronomist/Program Leader at the Crop Stress Research Laboratory, Department of Agronomy, Taiwan Agricultural Research Institute, Taiwan (ROC). He holds B.S. (1980) and M.S. (1982) degrees from the National Chung-Hsing University, Taiwan (ROC) and a Ph.D. (1990) from Texas A&M University (USA). His research interests include environmental agronomy, stress physiology, agrometeorology, and remote sensing technology (ground). Current research is focused on studies of the spectral characteristics in relation to nitrogen requirement, water deficiency, and pest infections (brown planthopper, leaffolder, leaf blast, and bacterial blight) in rice plants. Particular emphasis has been placed on establishing the mathematical relationships for quantitative estimation.
